# Electromagnetic Imaging Methods for Nondestructive Evaluation Applications

**DOI:** 10.3390/s111211774

**Published:** 2011-12-19

**Authors:** Yiming Deng, Xin Liu

**Affiliations:** 1 Imaging and Image Processing Laboratory, Department of Electrical Engineering, University of Colorado Denver, Denver, CO 80217, USA; E-Mail: xin.liu@ucdenver.edu; 2 Colorado Translational Research Imaging Center (C-TRIC), University of Colorado School of Medicine, Aurora, CO 80045, USA

**Keywords:** nondestructive evaluation, structural health monitoring, electromagnetic imaging, noninvasive imaging

## Abstract

Electromagnetic nondestructive tests are important and widely used within the field of nondestructive evaluation (NDE). The recent advances in sensing technology, hardware and software development dedicated to imaging and image processing, and material sciences have greatly expanded the application fields, sophisticated the systems design and made the potential of electromagnetic NDE imaging seemingly unlimited. This review provides a comprehensive summary of research works on electromagnetic imaging methods for NDE applications, followed by the summary and discussions on future directions.

## 1. Introduction

**Figure 1.**
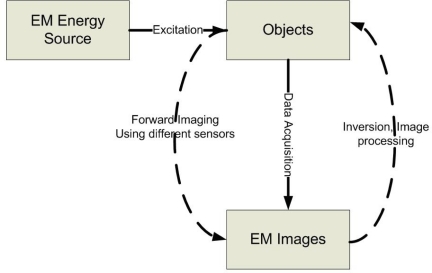
General schematic of electromagnetic NDE imaging system.

The development of imaging techniques for investigating physically inaccessible objects has been a topic of research for many years and have found widespread applications in the field of nondestructive evaluation (NDE) [1]. All electromagnetic (EM) methods in nondestructive evaluation involve Maxwell’s equations and cover a broad range of the electromagnetic spectrum, from static or direct current (DC), such as magnetic particle method, to high frequencies, e.g., X-ray and gamma-ray methods. Imaging, simply speaking, is the formation of images. Imaging science is concerned with the formation, collection, duplication, analysis, modification, and visualization of images. Electromagnetic NDE imaging is essential for detecting anomalies or defects in both conducting and dielectric materials by generating two-dimensional (2D) or three-dimensional (3D) image data based on the electromagnetic principles. A generic EM NDE imaging system can be simply represented as shown in Figure 1. For the forward imaging approaches, the excitation transducers usually couple the EM energy into the test objects, while the receiving sensors measure the response of energy/material interaction. Depends on different energy types and/or levels, various EM sensors/transducers can be used for a broad range of applications, e.g., eddy current imaging, microwave imaging, terahertz imaging, *etc*. After acquiring and storing the EM images, those data are passed through the inversion techniques block, which involves the object reconstruction, pattern recognition, *etc.* [2].

The usable electromagnetic frequencies for NDE purposes cover almost the entire EM spectrum, from DC to gamma radiation at the short wavelength end. However, this review article covers the imaging methods according to the different excitation EM frequencies only up to terahertz (*THz*). The reasons are two-fold: first, the optical NDE imaging with frequencies as low as in infrared range has been recently reviewed by another group [3]; second, the paper is intended to summarize and discuss the image modalities from the fields and waves perspective. As a result, the X-ray and gamma-ray NDE imaging techniques are not included here. Meanwhile, for non-EM methods, a heavily cited review article authored by Achenbach on quantitative NDE but focusing on acoustic imaging and applications was published in 2000 [4], which the readers can refer to for a complete understanding of NDE imaging methods.

## 2. Techniques Based on Different Electromagnetic Frequencies

The EM NDE imaging techniques covered in this review article include: direct current (DC) or static imaging methods, such as magnetic flux leakage imaging and impedance tomography imaging, to low frequency eddy current (EC) imaging that includes conventional EC imaging, EC sensor arrays, EC tomography and recently developed high resolution EC-based microscopy. Within the high frequency bands, microwave imaging, millimeter wave imaging and *THz* imaging are discussed. Some emerging methods like hybrid EM imaging, super-conducting quantum interference device (SQUID) imaging are also briefly summarized at the end of the paper. Overall, this paper is organized based on the different frequencies from longer wavelength end to shorter wavelength end those are applied in the EM imaging. Inverse problems in imaging such as image reconstruction, image characterization and analysis are also included in each section as key components of EM NDE imaging.

### 2.1. Static Electromagnetic Methods

In 1988 and 1990, Jiles at Center for Nondestructive Evaluation (CNDE), Iowa State University published two comprehensive and pioneering reviews about a variety of magnetic methods for NDE on ferromagnetic materials [5,6]. Magnetic particle inspection, magnetic flux leakage, leakage field calculations and eddy current inspection, including the remote field electromagnetic inspection method have been briefly discussed in his papers and he foresaw that with the upsurge of interest in that field, there must certainly be new magnetic methods awaiting development in the near future. In the past two decades, his statement has been validated as numerous electromagnetic imaging methods were developed while the ongoing research in this field still offers wide scope for future development and growth. In comparison with ultrasound, the instrumentation required in EM imaging is becoming far complicated than it used to be. However, the unique advantages that EM imaging has made this field promising, which is also the main driving force for the authors to review the current EM NDE imaging that are up to date through this article.

#### 2.1.1. Magnetic Flux Leakage Method

**Figure 2.**
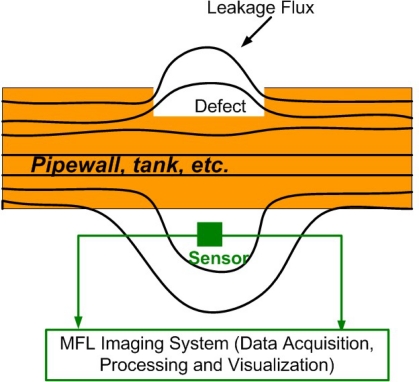
MFL inspection and imaging of gas pipelines.

Magnetic flux leakage(MFL) method is one important and widely used techniques in static eletromagnetic imaging methods. It is used for nondestructive evaluation of ferromagnetic objects and generates grey scale images that are representative of the integrity of those objects. Defective areas typically appear as bright regions in the image. The schematic of an MFL imaging system can be found in Figure 2. It clearly shows effects of induction on magnetic lines at discontinuity: surface leakage flux occurs at high magnetization level and with defects present, then gets picked up by the MFL sensors before passing to the imaging system for processing.

Since early 1990s, Udpa’s Materials Assessment group at Iowa State University (now Nondestructive Evaluation Laboratory at Michigan State University) were one of the pioneering groups in the US conducting MFL inspection and imaging research [7–10].

Their work mainly focus on defect characterization using MFL imaging data while the effect of variations in the test parameters associated with the experiment presenting. Mandayam and Udpa developed several novel but general techniques for deriving selective image invariants and performing invariance transformations to compensate for such variations. For example, wavelet basis functions can be used and typical magnetic flux leakage images obtained from finite element (FE) simulation of the pipeline inspection process are presented in their paper [7].

**Figure 3.**
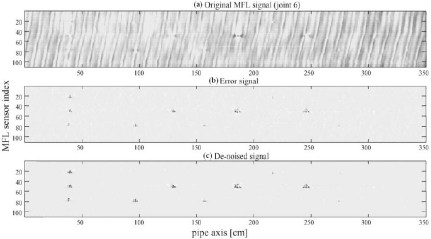
Results obtained from the application of the noise cancelation algorithm [8]. (**a**) Raw MFL image; (**b**) Output after SPN cancelation; (**c**) Final de-noised image.

In 2002, Afzal and Udpa introduced a new technique that employs wavelet based de-noising and adaptive filtering for detecting signals in MFL images, which were obtained from seamless pipes [8]. The proposed algorithm is computationally efficient and data independent. Field test imaging results before and after the seamless pipe noise (SPN) removal are shown in Figure 3. The stripe pattern can easily bury the useful information and significant improvement has been demonstrated according to the original MFL image and de-noised images comparison.

**Figure 4.**
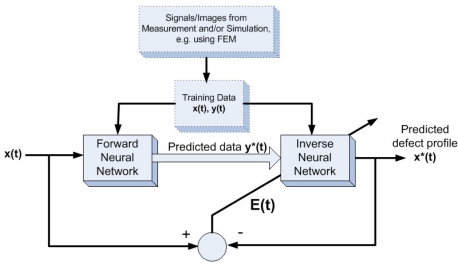
Feedback neural network configuration.

**Figure 5.**
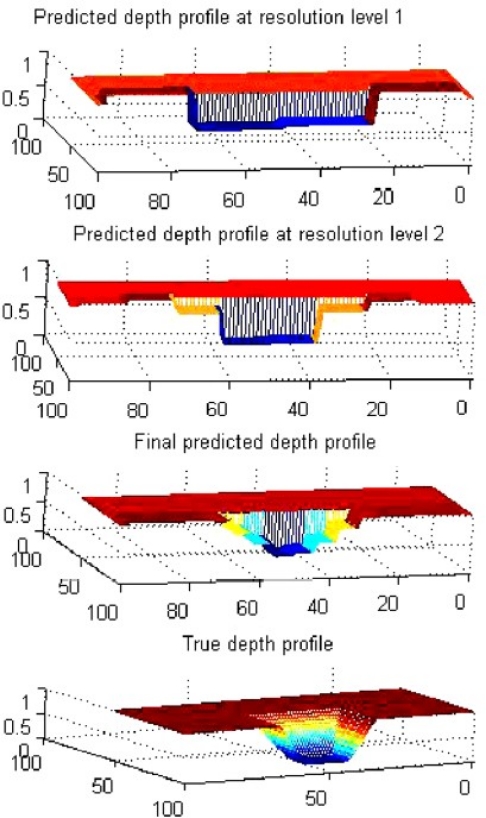
Image reconstruction results showing coarse to fine predication of the depth profile.

For image inversion techniques, the same group published two articles on MFL image reconstruction based on neural networks in 2002 and 2003 by Ramuhalli and Udpa [9,10]. In their papers, the radial basis function and wavelet basis function were first trained to approximate the mapping from the measured signal to the defect space, and the trained networks were used iteratively to estimate the defect profile. They extended their approach to the innovative *multi-networks* that are in feedback configuration and composed of a forward network and an inverse network. The schematic of the *multi-networks* are shown in Figure 4 with typical inversion results presented in [10]. Three-dimensional defect reconstruction for MFL imaging were further developed in 2006 by Joshi and Udpa based on the RBFNN approach with the coarse to fine results shown in Figure 5 [11].

Besides the efforts on those basis functions based forward and inverse methods, Haueisen *et al*. investigated both linear and nonlinear methods that include maximum entropy method (MEM), low resolution electromagnetic tomography (LORETA), *L*_1_ norm and *L*_2_ norm methods, to detect and characterize the MFL imaging data in early 2000s. Image reconstruction results using the above three methods were compared in their 2002 paper [12] concluding that the MEM, *L*_1_ norm and *L*_2_ norm methods usually performed well in MFL data inversion. However, in the authors’ opinion, both Udpa and Haueisen’s approaches suffer from the dependence on high and costly computational resources needed.

**Figure 6.**
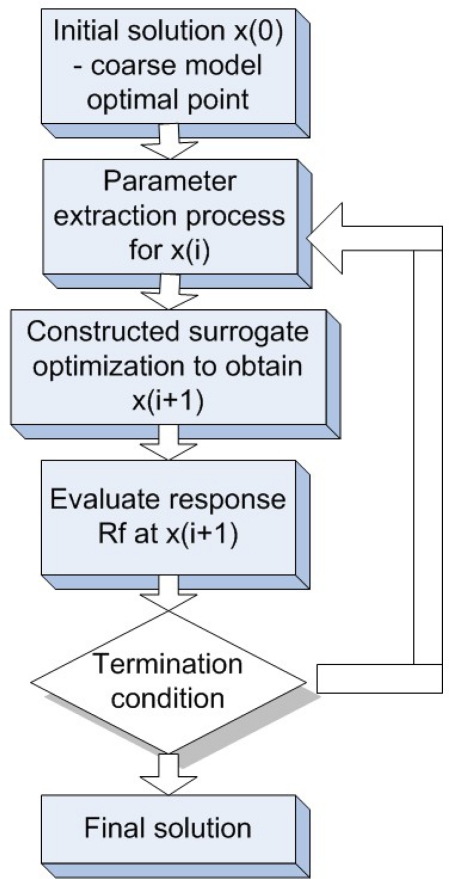
Flowchart of space mapping optimization.

Recently, space mapping methodology(SMM) was proposed by Amineh *et al*. for defect characterization in MFL imaging, which is efficient due to the optimization burden shifts from a computationally expensive accurate or fine model to a less accurate or coarse model with faster speed. The simplified flowchart of the space mapping method is shown in Figure 6, the detailed SMM approach can be referred to the paper published in 2008 [13]. The challenge to achieve both fast computation or image processing and better interpretation of physics remains an active research topic.

**Figure 7.**
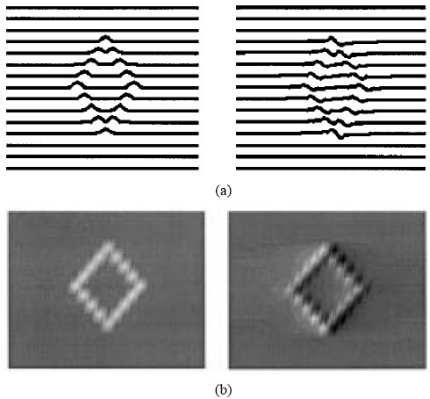
Composed images of the artificial grooved rhombic defect.

**Figure 8.**
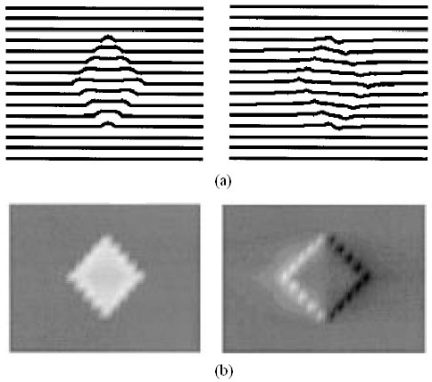
Composed images of the artificial hollowed rhombic defect.

Besides the image analysis and signal reconstruction for better interpretation and understanding of the MFL data, efforts on improving this magnetic imaging system itself have also been constant. In 2002, Parks proposed an optimum design to determine the size of the magnet in order to maximize the MFL signals and consequently generate superior MFL images [14]. The sensitivity of their optimum imaging system has been increased up to 200% and verified by measurement according to their findings. Their superior imaging results are shown in Figures 7 and 8. The authors do believe the next major breakthrough for MFL imaging should be initialized by development in innovative imaging instruments.

Most recently, a group in UK led by G.Y. Tian proposed to overcome the pitfalls of traditional MFL imaging by measuring the 3D magnetic field. Instead of the measurement of the two field components perpendicular to the testing surface (*z* axis) and parallel to the applied field (*x* axis), a high sensitivity three–axis magnetic field sensor was employed in their lab. Both finite element and experimental results demonstrated the merits of including additional information from the *y* axis. The 3D MFL imaging in Li and Tian’s work improved the defect characterization capabilities significantly, especially for irregular geometries. Both FE and experimental results were presented in 2007 [15].

Sophian *et al*. presented a new approach of imaging mechanism in 2006, termed as pulsed magnetic flux leakage (PMFL) method. Conventional MFL imaging techniques suffer in crack characterization such as sizing in the situations where defects take place on the near and far surfaces of the structures under inspection. Without introducing extra transducers or extra field components, like what Li and Tian proposed above, to overcome this problem, the PMFL clearly demonstrated advantages in terms of defect location and sizing by extracting features in time–frequency domain [16].

#### 2.1.2. Electromagnetic Tomography Imaging

Eggleston *et al*. published one of the pioneering papers in 1990 on EM tomography imaging, who developed the electric current computed tomography for defect imaging in metals [17]. A variety of clinical and nonclinical applications were developed since then and the imaging method is well known as electrical impedance tomography (EIT). EIT seeks the electrical conductivity and permittivity inside a body or structures, given simultaneous measurements of electrical currents and potentials at the boundary. However, human imaging using EIT has gained a lot of attentions and achieved relative successes over those NDE imaging applications. One of the most cited articles was published in 1999 by Cheney *et al*., in which a survey on EIT and its mathematical model was extensively discussed [18]. Borcea *et al*. reviewed the theoretical and numerical studies for the inverse problems of EIT [19] in 2002. Another excellent review article on the pitfalls, challenges and developments of EIT imaging was published by Lionheart *et al*. in 2004 [20], which is worth mentioning. Similar imaging method, such as electrical resistive tomography(ERT), was also introduced and summarized in 2002 by Kemnaa *et al*., (for details, see [21]).

**Figure 9.**
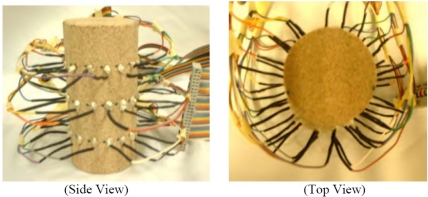
Side and top view of the electrode design.

**Figure 10.**
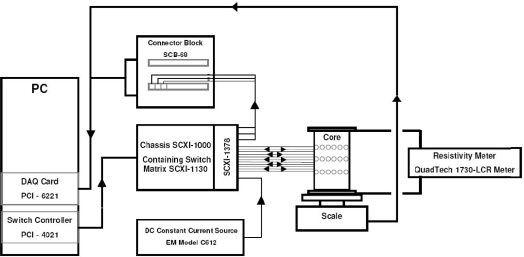
EIT system schematic developed at Stanford University.

In the authors’ point of view, the three-dimensional EIT imaging is still in its infancy for nondestructive evaluation purposes. Stacey *et al*. at Stanford University published a technical report in 2006 on 3D EIT imaging that provides estimates of reservoir saturation at multiple scales by determining the resistivity distribution within the subsurface [22]. Although their system is limited to specific applications, their initial experimental results are promising, which used a Berea sandstone core with 48 electrodes attached in three rings of 16. The side and top view of electrode design is shown in Figure 9 and the EIT imaging system schematic is illustrated in Figure 10. To summarize their system, the voltage potential field was measured by applying a direct current pulse across the core and measuring the voltage potential at all electrodes, essentially applying the 4–wire resistance technique over all electrodes in turn. The PC cycles through the sequence by measuring the voltage potential at every electrode before changing the current source electrodes. The current is supplied by the data acquisition (DAQ) card. The scale and resistivity meter are used to calibrate the EIT measurements by providing the actual saturation and resistivity [22]. EIDORS toolkit, which was developed for applications to nonlinear and ill-posed inverse problem, was utilized. Experiments conducted by Stacey *et al*. have indicated that 3D EIT is a viable technique for studying the displacement characteristics of fluids with contrasting resistivity and is capable of detecting displacement fronts in near real-time. Again, in our perspective, EM tomography imaging techniques have been underestimated in NDE community and should be exploited more in future. More discussion will be conducted in the last section of this paper.

**Figure 11.**
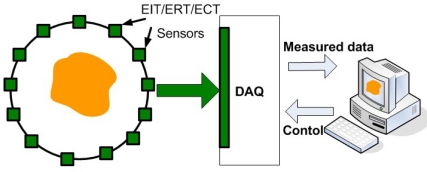
Typical ECT system with an eight electrode sensor, with sensing field boundaries shown.

In contrast to EIT and ERT, electrical capacitance tomography (ECT) imaging attempts to image the permittivity distribution of an object by measuring the electrical capacitances between sets of electrodes placed around its periphery. Yang *et al*. reviewed the existing image reconstruction methods for ECT, including linear back-projection, singular value decomposition, Tikhonov regularization, Newton–Raphson, iterative Tikhonov, the steepest descent method, Landweber iteration, the conjugate gradient method, algebraic reconstruction techniques, simultaneous iterative reconstruction techniques and model-based reconstruction [23]. Figure 11 shows a typical EIT/ERT/ECT system with an multiple electrode sensor.

**Figure 12.**
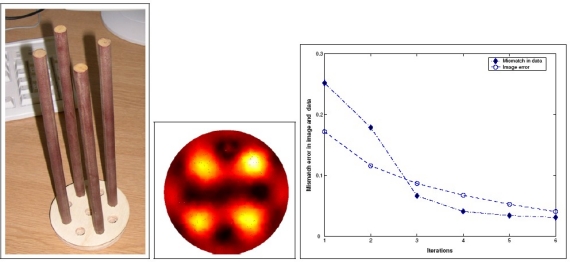
Reconstruction of four plastic rods shown in (**left**) from experimental data is shown in (**middle**). The normalized two norm of the mismatch error between measured and simulated capacitance is shown in (**right**).

In addition to Yang’s comprehensive review paper on ECT reconstruction, Soleimani *et al*. studied the nonlinearity of the inverse permittivity problem of ECT and implemented a regularized Gauss–Newton for nonlinear image reconstruction with adoption of finite element method(FEM) as the forward model solver [24], recently in 2005. The ECT results of four plastic rods is shown in Figure 12. Soleimani and colleagues presented a Helmholtz type Regularization Method for ECT reconstruction in 2010 [25]. More recent literatures on ECT development can be referred to [26,27] and [28].

The other advances in magnetic based imaging other than the increasing in sensitivity include but are not limited to that, for example in 2004, Knauss *et al*. reported a high resolution, non-contact magnetic based current imaging technology localizing high resistance defects in packages to within 30 *μm*, an order of magnitude better than time domain reflectometry [29]. They also applied this novel imaging technique on various applications, such as present and next generation semiconductor devices by introducing the very low magnetic field [30].

### 2.2. Quasi-Static Imaging Methods

This section reviews the electromagnetic imaging methods with low excitation frequencies: quasi-static imaging techniques such as conventional eddy current imaging. Several hybrid imaging techniques utilizing eddy current are also summarized here including the EC-based magneto-optic (MO) imaging and EC-based giant magnetoresistive (GMR) imaging.

#### 2.2.1. Eddy Current Imaging

Eddy current methods were initiated a few decades back and used extensively as one important electromagnetic NDE methods. Eddy current imaging is widely accepted as a nondestructive testing technique enabling efficient flaw reconstruction based on much richer and comprehensive data sets than the traditional Lissajous patterns obtained from a single EC scan [31]. Some of the pioneers, such as William Lord at Colorado State University in the 1980s, advanced both the theoretical and experimental aspects of EC imaging greatly [32–34]. In 1991, L. Udpa and S. S. Udpa co-authored a paper on neural networks based EC signals classification and the major contribution of their work was to introduce a rotation- and translation-invariant internal representation of the signals [35]. During the same year, Zorgati *et al*. published another pioneering paper on quantitative EC imaging of anomalies in conductive materials, which is the reflection mode diffraction tomography (DT) technique [36]. The applications of deterministic and stochastic quantitative inversion techniques to similar configurations were published later. Another early paper on EC imaging was published by Guettinger in 1993 [37].

**Figure 13.**
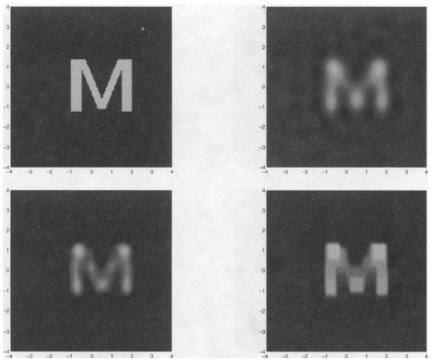
Numerical reconstructions from noisy synthetic data. On the top left is the true loss profile; on the top right is the profile obtained using least squares; and on the bottom row are the effects of adding positivity and total variation penalty.

Besides defect detection, material loss due to corrosion can also be imaged by eddy current and by assuming the linearized relationship between eddy current loop impedance change with the loss profile. Luong *et al*. developed a quantitative EC imaging system for corrosion detection and characterization in 1998 [38]. Numerical results using consistent data with noise are shown in Figure 13, which demonstrate the capability of the imaging system to accurately and quantitatively estimate the corrosion loss.

**Figure 14.**
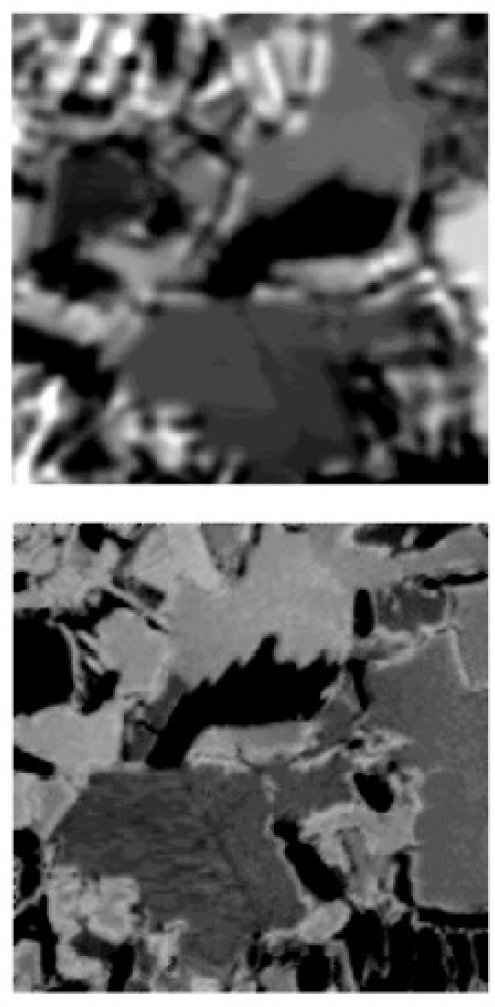
Comparison of eddy current and acoustic microscopic images of a coarse grained *Ti-6Al-4V* sample from nearly the same area of the sample.

**Figure 15.**
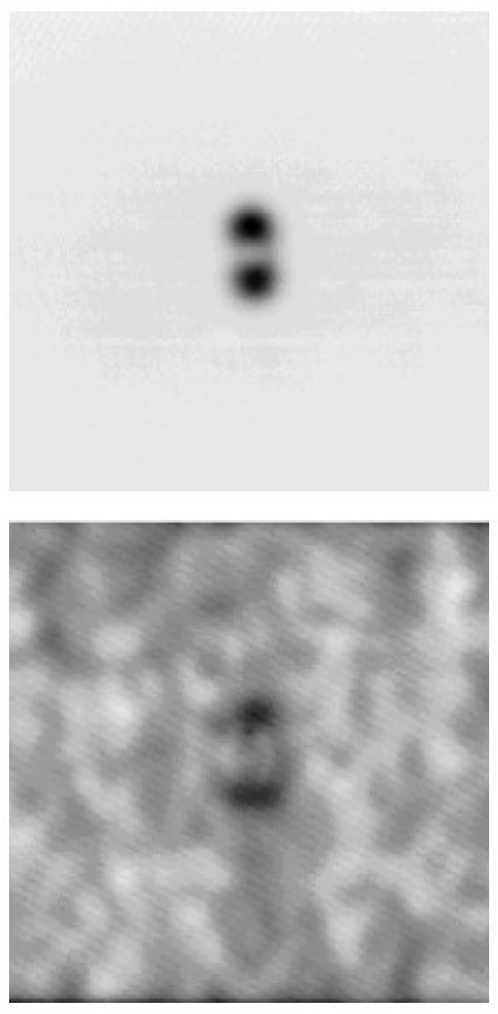
Eddy current images of small fatigue cracks in 2024 aluminum and *Ti-6Al-4V* samples.

Understanding the physics and physical limits of eddy current imaging is always important. From both theoretical and experimental approaches, Auld *et al*. [39] and Albanese *et al*. [40] published during the same year of 1999 on eddy current modeling to understand how the eddy current energy interacting with the materials. Both the forward problem and inversion techniques were covered in their work. Blodgett and Nagy *et al*. investigated the lateral resolution limits of EC imaging in 2000 with results shown in Figures 14 and 15 [41]. They performed comparison between eddy current microscopy and acoustic microscopy results, which demonstrated the feasibility of high resolution EC imaging.

Also it is worthwhile to mention that innovative EC imaging sensors were continuously developed in the past two decades, such as array geometry invented for reconstruction of 3D flaw images by Gramz *et al*. in 1994 [31], orthogonal coils EC transducer proposed by Grimberg in 2000 [42] and the EC magnetic induction tomography (MIT) imaging system by Soleimani in 2006 [43]. Similar to the EIT in the static EM imaging category, the MIT tries to image the electrical conductivity of the target based on impedance measurements, however by injecting energy from pairs of EC excitation and generating images from detection coils.

Recently, a circular EC probe array geometry was introduced by Abascal in 2008, for the applications of measuring the variations of impedance data collected close to the inner surface of the metal tube, which can further characterize the locations and shapes of defects, such as inner, outer and through-wall void flaws [44]. As the readers can tell, new EC sensors will be on a track of continuously development at both academic institutions and commercial sectors. One most recent development is the rotating magnetic field probe design at Michigan State University (MSU) that were presented at the Quantitative Nondestructive Evaluation (QNDE) conference held in Burlington, VT in 2011.

**Figure 16.**
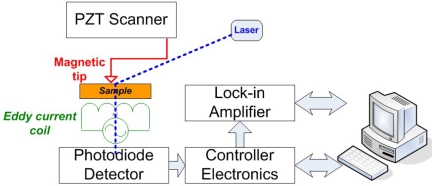
A general schematic of eddy current imaging setup using AFM.

**Figure 17.**
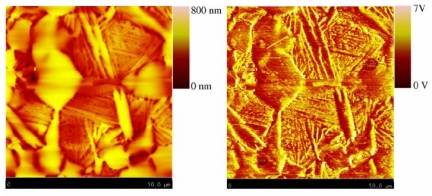
Topography and eddy current image obtained on Titanium alloy.

In 2008 and 2009, Nalladega *et al*. published an interesting paper and his doctoral dissertation [45], respectively, on a high resolution electrical conductivity imaging technique based on the principles of eddy current and atomic force microscopy (AFM). In the imaging system that he proposed, an electromagnetic coil is used to generate eddy currents in an electrically conducting material. The eddy currents generated in the conducting sample are detected and measured with a magnetic tip attached to a flexible cantilever of an AFM [46]. The contrast in the image was explained based on the electrical conductivity and eddy current force between the magnetic tip and the sample, where the spatial resolution of the eddy current imaging system was determined by imaging carbon nanofibers in a polymer matrix. The schematic of this high resolution eddy current microscopy is illustrated in Figure 16 with imaging results shown in Figure 17 [47].

#### 2.2.2. Pulsed Eddy Current Methods

Pulsed Eddy Current (PEC) Imaging was initially proposed during the early 1990s but bloomed during the past decade [48–50]. PEC imaging takes advantage of the broad frequency spectrum of an short excitation pulse in time domain over the conventional single frequency EC imaging.

**Figure 18.**
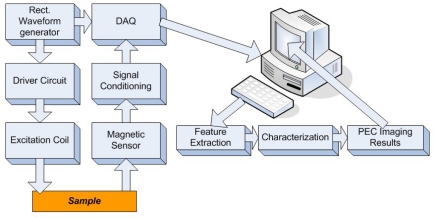
Block diagram of PEC imaging system.

The early pulsed eddy current sensors usually extracted defect information from the peak values and temporal profiles of the signals. Tian *et al*. proposed a prototype of pulsed eddy-current imaging with multiple sensors and used the principal component analysis (PCA)-based feature extraction that provides orthogonal information [51]. The schematic of a typical PEC imaging system is shown in Figure 18.

**Figure 19.**
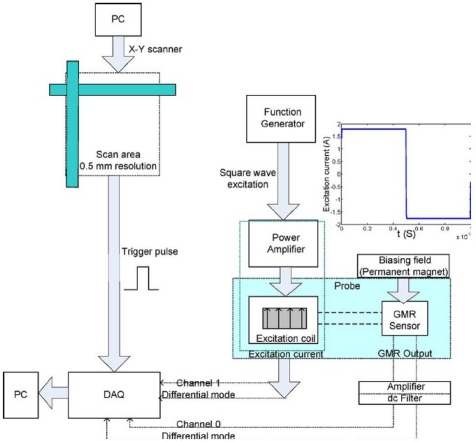
Schematic of the PEC-GMR imaging system.

**Figure 20.**
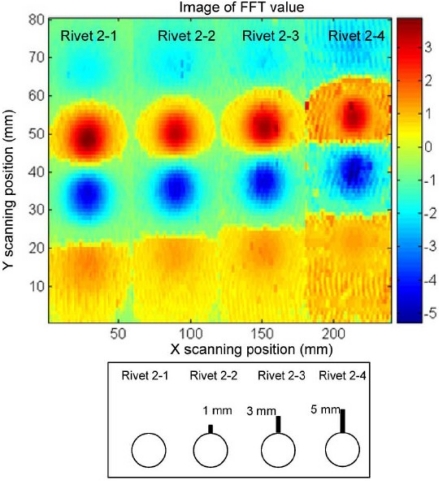
C-scan images derived from the transient GMR signals.

In 2010, the authors and their colleagues Yang *et al*. developed a novel PEC–GMR imaging technique by integrating the giant magnetoresistive field sensor for the detection and characterization of buried cracks in multiple layered structures [52]. The PEC–GMR system is illustrated in Figure 19 and typical imaging results shown in Figure 20.

**Figure 21.**
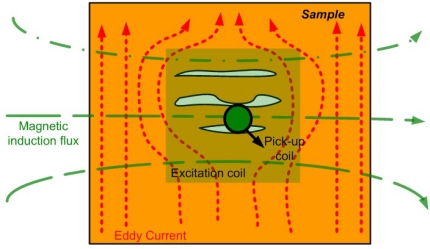
Top view of PET probe in direction of magnetic induction flux.

**Figure 22.**
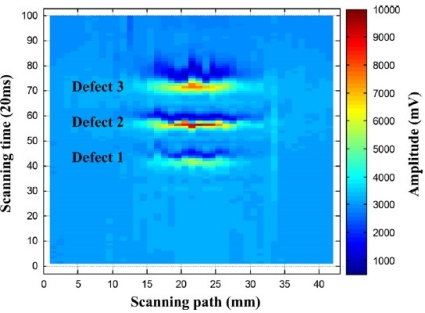
C-scan imaging results of three typical defects.

A different excitation coil structure with rectangular shape was proposed by He *et al*. in 2011 and various C–scan images were obtained for the buried subsurface defects. The top view of the PEC probe can be seen in Figure 21 and their sample PEC images were shown in Figure 22 for three different crack sizes [53].

#### 2.2.3. Eddy Current Magneto-Optic Imaging

**Figure 23.**
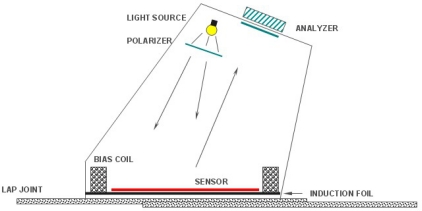
Schematic of the MOI system.

In order to achieve faster imaging speed with higher image resolution, several EC-based hybrid imaging techniques were developed. One of the most successful methods among them is the magneto-optic (MO) imaging technique invented by Shih and Fitzpatrick in the early 1990s [54]. The magneto-optic imager (MOI) is widely used in detecting surface and subsurface cracks and corrosion in aircraft skins. The instrument provides analog images of the anomalies based on eddy current induction techniques and an MO sensor using the Faraday rotation effect. The schematic of the EC–MOI system is shown in Figure 23.

The merits of the MOI that make it attractive include rapid and large-area inspection, insensitivity to liftoff variations, and easy interpretation in contrast to the complex impedance data of conventional eddy current inspections [55]. Fan and Deng *et al*. developed a real-time aircraft rivet imaging, crack detection and classification system implemented on a TMS320C6000 DSP platform in 2006 and demonstrated at Kuka Robotics in Detroit, 2007. Their system can not only reduce the detection variability from inspector to inspector but also have the capability of fully automated image analysis, such as segmentation, enhancement (noise removal), quantization and classification [56]. A quantitative basis for MO image processing and characterization was established by Deng *et al*. during the same year by introducing the *skewness functions*. Meanwhile, to understand the MO imaging physics, a numerical simulation model that produces quantitative values of the magnetic fields associated with induced eddy currents interacting with structural defects using 3D FEM simulation was presented by Zeng *et al*. [57], which is an essential complement to the instrument development process. This eddy current based hybrid technique became a great success. The dynamic collaborative research team among government, industries and academia including the authors was awarded the 2005 FAA-ATA Better Way Award because of their contributions in the MOI technique. This state-of-the-art system can be used by mounting the MO imager on a robot for fully automated scanning.

**Figure 24.**
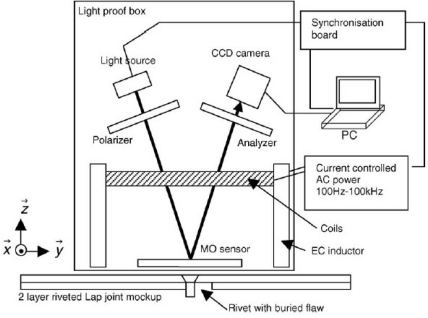
Schematic of the LMOI.

**Figure 25.**
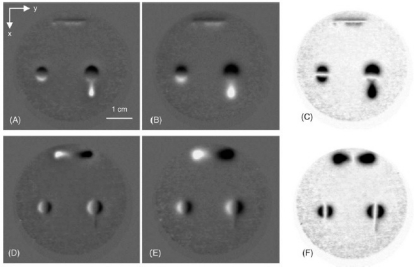
EC-MO images of a two layer riveted lap joint.

In 2006, one similar system named linear MO imager (LMOI) was patented in Europe by Joubert *et al*., which consists of the combination of a dedicated MO sensor featuring a linear and hysteresis-free magnetization loop, used with an original image acquisition system based on a stroboscopic approach, and a specific high sensitivity eddy current inductor [58]. The first schematic of the LMOI integrated prototype is shown in Figure 24, which is similar to the setup of the pioneering MOI system by Shih and Fitzpatrick. The field of view of Joubert’s LMOI system, in contrast to the US version, is circular as imaging results shown in Figure 25. This research group published another two articles in 2009 and 2010 for characterization of subsurface defects in aeronautical riveted lap joints using a multi-frequency imaging setting [59,60]. Further improvement on magneto-optic based imaging methods explored new sources of optical energy. Instead of using polarized light, Cheng *et al*. adopted laser and combined with MO thin films technology to achieve an enhanced MO imaging system in 2007 [61].

#### 2.2.4. Eddy Current Magnetoresistive Imaging

Another hybrid EC imaging utilized the Nobel-winning discovery, giant magnetoresistive effect, and measured the 3D magnetic field generated by the eddy current perturbation directly. The use of giant magnetoresistive (GMR) sensors for electromagnetic imaging in nondestructive evaluation has grown considerably in the last few years. A key advantage of GMR sensors is a flat frequency response extending from DC to hundreds of MHz, making them particularly attractive for low-frequency and multi-frequency eddy current detection [62].

**Figure 26.**
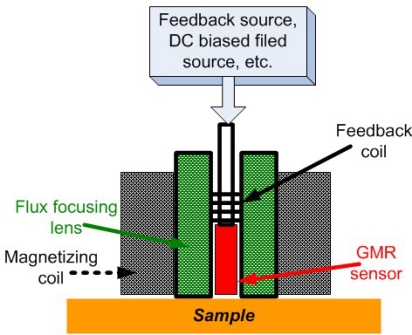
Schematic diagram of GMR-based self-nulling probe with active feedback.

For EC imaging, there is always a trade-off between the penetration depth due to *skin depth effect* and better image resolution that is directly related to frequency. In particular, the low frequency sensitivity of the GMR provides a practical means to perform electromagnetic inspections on thick layered conducting structures. Wincheski *et al*. at NASA Langley Research Center incorporated a commercially available GMR sensor into the self-nulling probe and their research showed that this imaging set up can greatly enhance the low frequency capabilities of the imaging device. By combining with image processing, their system has resulted in a greatly improved signal-to-noise ratio (SNR) for very deeply buried flaws in conducting materials [63]. Figure 26 shows an illustration of their system schematic.

If the readers want more background knowledge on magnetoresistive physics, one excellent review article on magnetoresistive imaging sensors was published by Jander in 2005, in which the physical principles, manufacturing process, and performance characteristics of the three main types of MR devices, anisotropic magnetoresistance (AMR), giant magnetoresistance (GMR) and tunneling magnetoresistance (TMR) are thoroughly discussed [62].

**Figure 27.**
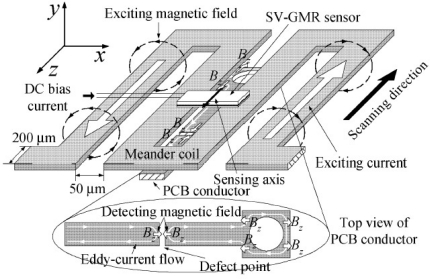
Schematic of SV-GMR based ECT probe.

In the year of 2006, Yamada *et al*. proposed a needle type GMR imaging technique, SV–GMR system and the applications of this new sensor including the inspection of bare printed circuit board and the measurement of the density of magnetic fluid injected in living body for the hyperthermia treatment [64]. The SV–GMR imaging system schematic is shown in Figure 27. Singh and Raj *et al*. developed another novel imaging system involved both GMR sensors and MFL principles for detection of various near side notches and far side notches in a 12 mm thick carbon steel [65]. At NDE lab of Michigan State University, a flexible and efficient real-time GMR imaging system for nondestructive evaluation of aircraft was developed by Nair *et al*. with several advantages over the past prototypes [66]. The reader can find other similar types of EC-GMR system that were developed within the last decades, for example the system by Postolache *et al*. published in 2008 [67] or Tsukada system in 2006 [68], *etc*.

Due to the low operating frequency and subsequent noisy signals, the EC–GMR image processing and analysis is always critical and challenging. Numerous efforts have been put into this problem, and in 2009, Deng *et al*. introduced the optimum detection angle (ODA) to combine the in-phase and quadrature components of GMR signals, enhanced the GMR image data by over ten orders of magnitude in SNR [69]. Kim *et al*. developed a PEC–GMR imaging platform in 2010 and proposed a principle component analysis (PCA) based feature extraction and classification algorithm for those data [70], which took advantage of both pulsed excitation and sensitive GMR sensors. Most recently, Zeng *et al*. published several quantitative metrics for characterizing EC–GMR images and the improvement in probability of detection(POD) was clearly demonstrated [71].

#### 2.2.5. Other Low Frequency EM Imaging Sensors

Other EM imaging sensors that fall into this low frequency category include, but are not limited to, magnetic induction tomography (MIT), giant magneto-impedance (GMI) imaging, which will be briefly introduced in this review article. MIT applies a magnetic field from an excitation coil to induce eddy currents in the material, and the magnetic field is then detected by sensing coils [72]. Griffiths *et al*. discussed the physics of MIT as a fascinating and new imaging modality for both industry and medical imaging, also the challenges in acquiring good quality MIT data in 2001.

**Figure 28.**
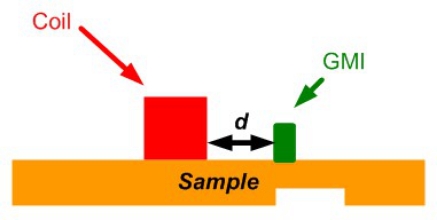
Schematic of the GMI based probe.

Vachera *et al*. developed a high sensitivity imaging sensor based on GMI effect that combines good sensitivity performances at low frequencies and small size of sensors in 2007 [73]. A simple configuration of GMI imaging is shown in Figure 28.

### 2.3. High Frequency Time Varying Imaging Methods

High frequency time-varying EM imaging methods are discussed in this section. In contrast to the static and low frequency EM imaging modalities, they have their unique advantages and specific applications.

#### 2.3.1. Microwave Imaging

**Figure 29.**
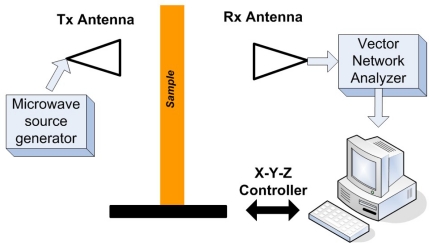
A typical microwave imaging system setup.

For the past a few decades, the tremendous advances of microwave NDE imaging is clearly one proof of the importance of EM imaging within this high frequency band. Several articles in the late 1980s and early 1990s foresaw the potential and a growing field of applications in microwave sensing, especially for nondestructive evaluation [74–76]. An excellent review on this topic was given by Zoughi *et al*. in the year of 2007 [77]. Examples of the state-of-the-art microwave imaging for various applications, such as inspection of Carbon Fibre Reinforced Polymer (CFRP) composite laminate strengthened structures, detection and evaluation of corrosion and precursor pitting under paint, *etc.*, were covered in this article. However, Zoughi stated that the inadequate commercial availability of microwave systems for NDE purposes has limited its more extensive implementation. The authors do believe and expect microwave imaging to be one of the most dominant and versatile NDE imaging techniques in the near future. A typical but simple microwave imaging system setup can be seen in Figure 29 [78].

In 1995, Diener *et al*. studied an imaging system with an open-ended waveguide at the frequency of 30 *GHz* for evaluation of dielectric materials. He compared the results obtained from microwave energy with the more prominent NDE methods at that time such as ultrasonics, X-rays and thermal waves and demonstrated the great performance of microwave imaging [79]. Another driving force for this technique lies in the biomedical applications and clinical imaging needs, for example, the breast cancer imaging and biological tissue characterization [80] utilize the microwave energy extensively. These medical related literatures will not be elaborated in this paper but are worth mentioning since it is another active research field of microwave imaging nowadays.

Another major application of microwave imaging is to determine the shape and location of an buried object or defect from the measurements of the field scattered by the object or defect. Underground object detection research has undergone a long way with numerous applications. Ground penetrating radar (GPR) techniques have been thoroughly studied and developed. Belkebir *et al*. proposed a microwave imaging system and tested two different reconstruction algorithms, a Newton–Kantorovich (NK) method and the modified gradient (MG) method by comparing their effectiveness and robustness [81]. In 1999, Tabib-Azar *et al*. imaged and mapped material non-uniformities and defects using microwave generated at the end of a micro-stripline resonator with 0.4 *mm* lateral spatial resolution at 1 *GHz*. They introduced a novel sensor called evanescent microwave probe (EMP) and demonstrated the overall capabilities of EMP imaging techniques as well as discussed various probe parameters that can be used to design EMPs for different applications [82].

**Figure 30.**
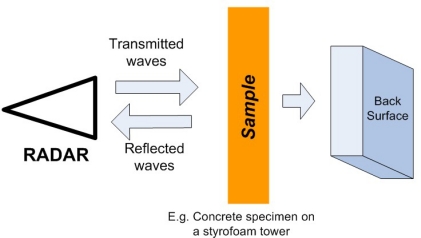
A typical radar imaging setup.

Critical infrastructure monitoring and inspection is always a challenging problem. Radar imaging has become a powerful and effective tool for the nondestructive testing of concrete structures. Weedon *et al*. proposed a step-frequency radar imaging system in 1994 [76]. An advancement of the method can be achieved through the understanding of the interaction between electromagnetic waves and concrete, and the identification of optimum radar measurement parameters for probing concrete [83]. Figure 30 is a simple radar imaging experimental system setup. Rhim *et al*. developed a wide band imaging radar to obtain 2D and 3D imagery of concrete targets.Three different types of internal configurations were imaged. For the determination of optimum parameters after systematic radar measurements, they found that 2 to 3.4 *GHz* waveforms are adequate for the concrete thickness measurement, 3.4 to 5.8 *GHz* waveforms are adequate for the detection of delamination, and 8 to 12 *GHz* waveforms are adequate for the detection of inclusions embedded inside concrete [83]. Rhim, in the year of 1998, also published the concrete materials characterization results using electromagnetic frequencies from 0.1 to 20 *GHz* that will serve as a basis in applying wide band microwave imaging techniques for NDE of concrete using radar [84].

**Figure 31.**
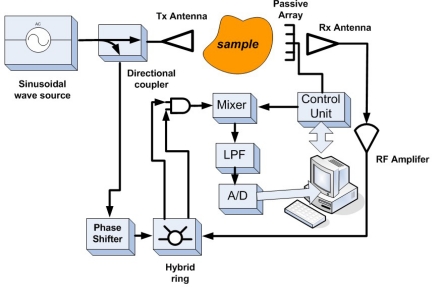
Modulated scattering microwave imaging system schematic.

In 2004, Pastorino summarized the development of efficient inverse scattering based procedures for electromagnetic imaging at microwave frequencies, especially for 2D tomographic imaging approach. He also introduced the modulated scattering technique, which is a promising technique strongly related to electromagnetic scattering concepts [78]. The modulated scattering system schematic is shown in Figure 31.

**Figure 32.**
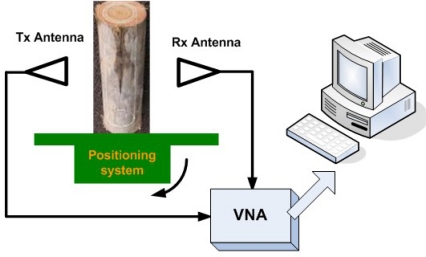
Block scheme of Pastorino microwave imaging system published in 2007.

**Figure 33.**
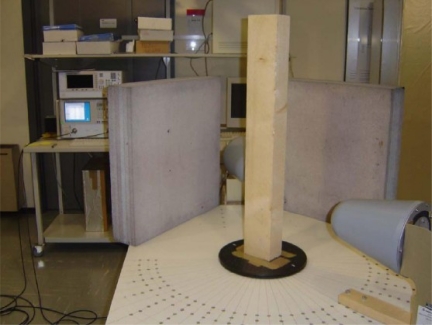
Pastorino microwave imaging system: illumination and measurement part of the experimental setup.

In [85], Pastorino extensively reviewed the stochastic optimization methods applied to microwave imaging with various imaging modalities considered, such as tomography, buried object detection and borehole sensing. Pastorino *et al*. also presented an experimental setup based on interrogating microwaves to obtain images of the cross section of dielectric cylinders. Both experimental results and numerical validations have been conducted. Figures 32 and 33 show the system schematic and experimental setup, respectively.

In the years of 2003 and 2004, Caorsi *et al*. proposed a hybrid genetic algorithm (GA) based microwave imaging procedure for detecting defects in dielectric structures by pre-computing the Green’s function for the configuration without defects and consequently saving significant imaging and reconstruction time [86,87], which was considered as a big improvement in microwave imaging inversion.

**Figure 34.**
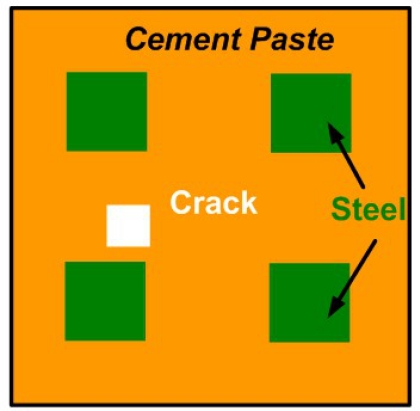
Civil structure model.

Similar types of efforts were carried out by Benedetti *et al*., who developed an innovative inversion procedure based on the use of GA and on the Sherman–Morrison–Woodbury (SMW) matrix inversion method [88,89] with the testing structure shown in Figure 34. Also, Massa *et al*. from the same group presented the improved tomographic microwave imaging approach based on the use of the SMW updating formula for electric field computation with applications in civil structures. Donelli and Massa *et al*. in 2005 developed another innovative stochastic algorithm called the particle swarm optimizer (PSO) for the solution of microwave inverse scattering problem [90]. In 2011, this group tried to solve the 2D inverse scattering problem by probing the unknown scenarios with TE and TM waves with multi-zooming approach [91]. Besides those literatures, there is another excellent technical article published in 2006 by Langenberg *et al*., who tried to unify the theory of electromagnetic, acoustic and elastic wave fields for imaging purposes with examples of bridge NDE [92].

Zoughi *et al*. conducted pioneering research in microwave imaging at the Missouri University of Science and Technology (known as University of Missouri–Rolla before) and he published a review paper in 2008 on the near-field microwave imaging demonstrating the capabilities of EM imaging for detecting cracks and evaluating their various dimensional properties including determining a crack tip location accurately [93] at time varying frequencies. Recently, Wu *et al*. presented the development of an experimental microwave tomography system intended for oil and gas flow measurements in 2009 [94].

#### 2.3.2. Millimeter Wave Imaging and Terahertz Imaging

Millimeter wave imaging or terahertz (THz) imaging has drawn more and more attention in recent years. The implementation of THz imaging for nondestructive evaluation shows great promise in 2D and 3D non-contact inspection of non-conductive materials such as plastics, foam, composites, ceramics, paper, wood and glass [95]. For a review of these high frequency NDE imaging techniques, the readers can refer to the paper by Kharkovsky *et al*. published in 2007 [77].

**Figure 35.**
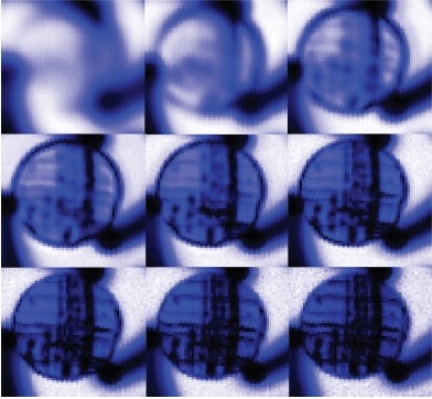
THz transmission images in 0.1 THz bandwidths from 0.1–0.2 THz through 0.9–1.0 THz. Note the improvement in spatial resolution with increasing THz frequency.

Similar to the efforts in high resolution EC imaging methods, Hor *et al*. in 2008 examined the cork’s surface and interior using this short wavelength and achieved roughly 100 to 300 *μm* resolution for the presence of voids, defects and changes in grain structures [96]. Their results can be seen in Figure 35.

**Figure 36.**
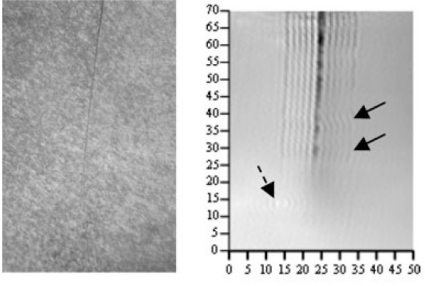
Picture of the fatigue crack obtained with a microscope (left) and the 90 GHz image (right) of the crack obtained at standoff distance of 0.8 mm. Solid arrows show the indication of crack non–uniformities and dash arrow shows the indication of pitting.

Zoughi *et al*. have successfully applied millimeter wave techniques on nondestructive detection and evaluation of stress-induced fatigue cracks in metals working in several critical environments including surface transportation (steel bridges, railroad tracks, railroad car wheels, *etc*.), aerospace transportation (aircraft fuselage, landing gears, *etc*.) and power plants (steam generator tubings, *etc*.) [93]. See Figure 36 for the comparison between the images obtained from microscope and the 90 *GHz* microwave imaging system. In 2009, Kharkovsky, who works with Zoughi, evaluated the efficacy of near-field millimeter-wave NDE techniques, using open-ended flange-mounted rectangular waveguide probes [97]. Because of the ability to penetrate through dielectric substances, Kemp *et al*. published the development of a millimeter and sub-millimeter continuous wave system for imaging corrosion pitting, structural defects and beat damage in common aircraft materials such as aluminum and polyamides in 2010 [98].

**Figure 37.**
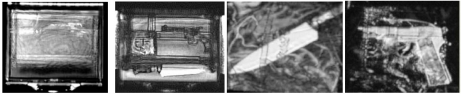
Reflection terahertz images.

On the other hand, for polymer materials, Beckmann *et al*. proposed to use THz frequencies from 0.05 to 2 *THz* to detect flaws in those materials like degradation areas in polymer pipelines, moisture distributions and de-laminations in composites used in aircraft industry [99]. In 2005, Zimdars *et al*. reported on the applications of a transmission and reflection reconfigurable large area time domain THz imager for homeland security and NDE applications [95,100]. See Figure 37 for their results of co-linear THz reflection imaging. Other imaging system development effort includes: a compact sub-THz imaging system that was presented by Oyama *et al*. for the application of inspecting timbers, concrete and ceramic tiles [101]. The most recent progress in general THz science and technology can be found in the review article authored by the researchers at RPI [102] and in another outstanding review paper by Bogue *et al*. in 2009 that provided a detailed insight into the present state of THz imaging [103].

### 2.4. Other EM Imaging Methods and Inverse Problems

It is neither a possibility nor our intention to complete an exhaustive search on all the EM imaging methods literatures for NDE applications. However, a comprehensive coverage of most of the major EM NDE imaging methods is our objective for this article and in this paragraph, we briefly introduce several other EM imaging methods that are important to NDE community, such as: the super-conducting quantum interference device (SQUID) imaging for NDE [104–106]; magneto-acoustic imaging [107,108]; millimeter acoustic imaging [109]; microwave induced thermoacoustic imaging [110]; and general EM tomography imaging techniques [111–115]. The inversion of EM NDE imaging is a separate but very comprehensive topic. If interested, the readers can refer to the following articles for more information [116–121].

## 3. Summary and Conclusions

A comprehensive and up-to-date review of electromagnetic imaging methods for NDE applications has been conducted in this article. The recent advances in sensing technology, hardware and software development dedicated to imaging, image processing and material sciences have greatly expanded the application fields, sophisticated the systems design and made the potential of electromagnetic NDE imaging seemingly unlimited. Like the research and development in other imaging techniques, there are always trade-off and hurdles for us in achieving both higher image resolution/SNR and lower noise, faster image acquisition and reasonably good image quality, *etc*. It is believed that the emerging technologies in computer engineering will significantly impact the EM NDE imaging development. For instance, by introducing the graphics processing units (GPU), it will not only redefine the computational related works for many imaging methods we mentioned above, but also push the development of imaging sensors forward through parallel data processing, fast image acquisition, enhancement and compression. The authors foresee that there is great potential and future development in the field of EM imaging for NDE and SHM applications, including both active and passive sensors.
